# Neuroplasticity Across the Autism–Schizophrenia Continuum

**DOI:** 10.3390/biomedicines13112695

**Published:** 2025-11-02

**Authors:** Evangelia Kesidou, Nikolaos Mitsoudis, Olympia Damianidou, Charilaos Taloumtzis, Marianna Tsakiridou, Eleni Polyzoidou, Eleni Grigoriadou, Christos Bakirtzis, Evangelia Spandou, Constantina Simeonidou

**Affiliations:** 1 Laboratory of Physiology, Faculty of Medicine, Aristotle University of Thessaloniki, 54124 Thessaloniki, Greece; kesidoue@auth.gr (E.K.); espandou@auth.gr (E.S.); ksymeoni@auth.gr (C.S.); 2 Laboratory of Experimental Neurology and Neuroimmunology, 2nd Department of Neurology, AHEPA University Hospital, Aristotle University of Thessaloniki, 54636 Thessaloniki, Greece; nickmitsoudis@gmail.com (N.M.); dolympia@auth.gr (O.D.); gastrogr@gmail.com (C.T.); marianniye@mail.com (M.T.); elpolyz@auth.gr (E.P.); helengrigo@gmail.com (E.G.)

**Keywords:** neuroplasticity, Autism spectrum disorder, Schizophrenia, neurodegeneration, neurorehabilitation

## Abstract

Plasticity is a fundamental property of the brain that enables the nervous system to respond appropriately to internal and external stimuli. It primarily involves changes at the synaptic level, mediated by a wide array of molecules, ultimately leading to cognitive and behavioral changes. This review critically contrasts the developmental timing and mechanisms of plasticity in Autism spectrum disorder (early hyperplasticity and excitation–inhibition imbalance) versus Schizophrenia (adolescent overpruning and NMDAR hypofunction) and evaluates evidence for interventions that harness plasticity to improve cognitive and behavioral outcomes. Preclinical and small clinical studies suggest that interventions targeting plasticity-related pathways may improve specific cognitive and behavioral domains. However, effects appear to be symptom-domain-specific and protocol-dependent and larger randomized controlled trials are needed to confirm efficacy. Cognitive remediation for Schizophrenia has been associated with improved executive function and increased hippocampal volume, while virtual reality-based training for Autism spectrum disorder has shown gains in attention and planning skills. By highlighting both molecular mechanisms and therapeutic strategies, this review aims to provide an integrated perspective on how plasticity-targeted interventions could be optimized across neurodevelopmental and neuropsychiatric disorders.

## 1. Introduction

Neural plasticity—the ability of the brain to adapt through changes in synaptic strength, number, pruning and neurogenesis—underlies learning, cognition and behavior [1]. Non-synaptic plasticity, including changes in excitability, dendrites and glial function, also shapes neural circuits. While both Autism spectrum disorder (ASD) and Schizophrenia (SCZ) involve disruptions in these mechanisms, they diverge in timing and pattern: ASD shows early hyperplasticity and excitation–inhibition imbalance, whereas SCZ is characterized by adolescent overpruning and NMDAR hypofunction. The concept of neuroplasticity may also be perceived as the balance between stability (homeostasis) and change, and the balance between long-term potentiation (LTP) and long-term depression (LTD) that strengthens and weakens, respectively, the connections of presynaptic and postsynaptic neurons [2].

Many studies in neurosciences have focused on the concept of synaptic plasticity through the investigation of nervous system development, learning and memory, responses to stimulations and recovery from central nervous system injuries [1]. Impaired mechanisms of synaptic plasticity seem to be involved with several neuropsychiatric disorders [3]. Therefore, further research is required to clarify the contribution of neural plasticity to both the normal and pathological organization and functioning of the nervous system.

In the early stages of brain development, there is a rapid period of synaptogenesis, neuronal differentiation and synaptic pruning, which gives rise to the basis of functional neural circuits [4,5,6,7,8,9]. These “critical windows” for widespread neuroplasticity are differentially altered regarding the various disorders: For example, in the case of ASD early postnatal synaptogenesis may be excessive, leading to hyperconnectivity and an imbalance of excitation–inhibition, while the SCZ disorders are associated with excessive neuronal pruning during adolescence, decreasing connectivity and accompanying cognitive deficits. Emphasizing these divergent windows provides a framework for understanding disorder-specific plasticity mechanisms. Building on these observations, this review advances a developmental-shift model of neuroplasticity across ASD and SCZ, conceptualizing both within a continuum of timing- and circuit-specific dysregulation that may inform future plasticity-targeted interventions.

## 2. Methods

We searched PubMed and Scopus databases up to the 31 August 2025 using the terms neuroplasticity, ASD, SCZ and rehabilitation interventions. Included studies encompassed human, animal and iPSC models reporting molecular, structural, or functional plasticity outcomes; excluded were non-English articles, case reports, or studies lacking relevant data.

## 3. Molecular Pathways of Plasticity

Among the key modifying mechanisms of neuronal networks is long-term synaptic plasticity that includes LTP and LTD, as well as short-term synaptic plasticity (STP) [3]. During LTP, high-frequency stimuli can strengthen the synapses, whereas low-frequency stimuli can weaken them during LTD [10]. Occurring synaptic changes are followed by morphological remodeling of dendrites and axons. More specifically LTP is accompanied by a characteristic increase in the size of dendritic spines [11,12], or their number during motor skill memory acquisition [13], while their size decreases in LTD [1,14]. Sensory stimuli and learning, also affect brain plasticity by inducing changes in the cortex after practice [15].

A multitude of mechanisms is involved in synaptic plasticity with most of them relying primarily on *N*-methyl-d-aspartate Receptors (NMDARs) and α-amino-3-hydroxy-5-methyl-4-isoxazole-propionic Acid Receptors (AMPARs) [1]. The increase in synaptic strength during LTP depends on the increase in AMPARs on the postsynaptic membrane, whereas maintenance of LTP, which is important in establishing new memories and long-term memory, requires protein synthesis [12,16], development of new dendritic spines, morphological remodeling and growth of synapses [3]. Alterations in the number or subunits of synaptic NMDARs may affect the level of Ca^2+^ influx in the postsynaptic neuron and, thus, the threshold of synaptic plasticity mechanisms induction [17,18]. In SCZ, DISC1 and NRG1 variants can impair NMDAR signaling and downstream dendritic remodeling, reducing LTP efficiency and contributing to cognitive deficits. In ASD, FMRP loss and dysregulation of mTOR/PI3K/SHANK3 pathways disrupt AMPAR trafficking and dendritic spine morphology, promoting hyperplasticity and aberrant circuit connectivity.

The most studied LTP mechanism in mammalian brains is the voltage dependent NMDAR LTP [19]. During this mechanism, activation of Mg^2+^-blocked NMDARs by glutamic acid results in Ca^2+^ influx into postsynaptic dendritic spines [17]. High Ca^2+^ concentrations activate LTP signaling cascades that involve kinases, such as CaMKII [17], which contribute to the increase in functional AMPARs [20]. Other receptors, such as β-adrenergic receptors (βRs), rely on cyclic AMP (cAMP) and on the activation of protein kinase A (PKA) which phosphorylates AMPAR subunits, allowing its incorporation into the membrane. The late phase of LTP, during which gene expression takes place, involves Mitogen-Activated Protein Kinase 2 (MAPK2 or ERK2) with MAPK2–3 (or ERK1–ERK2) being activated by the RAS cascade–RAF–MEK (MAPK–ERK) through Receptor Tyrosine Kinase-RTKs [21,22].

Other forms of plasticity include NMDAR-dependent LTD, which occurs due to weak activation of NMDARs possibly due to lower Ca^2+^ levels postsynaptically in comparison to LTP. Moreover, dephosphorylation of synaptic substrates, such as AMPARs [19], following the activation of serine/threonine phosphatases pathways limits their strength and the size of dendritic spines, underlining the importance of AMPARs in synaptic remodeling [23]. LTD can, also, be induced by activation of metabotropic glutamate receptors (mGluRs), as it has been observed in the cerebellum, hippocampus and neocortex [19]. In synapses of the central nervous system that release glutamic acid and GABA, a small influx of Ca^2+^ postsynaptically can initiate the synthesis of endocannabinoids that in cases of overproduction can induce LTD [17,24]. In SCZ, impaired NMDAR signaling and excessive pruning may exaggerate LTD, contributing to synaptic weakening and cognitive deficits. In ASD, dysregulated mGluR-LTD and endocannabinoid signaling, often downstream of FMRP or SHANK3 dysfunction, may disrupt the balance of excitatory/inhibitory networks, reinforcing hyperconnectivity and aberrant plasticity (Table 1).

Additional concepts include homeostatic plasticity, which refers to compensatory alterations to changes in neuronal activity due to LTP and LTD in order to maintain homeostasis in space and time [1,3]. Metaplasticity, often referred to as “the plasticity of plasticity”, represents a higher-order form of synaptic plasticity in which prior synaptic activity modifies the subsequent direction or magnitude of plastic changes. This mechanism supports the preservation of a dynamic range of synaptic activity and regulates their adaptability to environmental influences [3]. Finally, STP, that lasts from milliseconds to minutes, is important for short-term responses to sensory stimuli, small changes in behavior and short-term memory. Typically, it is triggered by bursts of activity leading to presynaptic calcium build-up and changes in neurotransmitter release [3]. Disruptions in STP mechanisms, including presynaptic Ca^2+^ handling, have been linked to cognitive and sensory processing abnormalities in both ASD and SCZ.

Impairments in the physiological mechanisms of brain development and especially in mechanisms that affect synapses are associated with developmental disabilities that impact children’s mental, behavioral and physical functioning. The vast majority of changes during brain development take place until the fifth year of childhood, while it receives the most stimuli, a period that is critical for the proper development in other aspects like vocational attainment and education [25]. Functional disruptions during this period could eventually lead to sensory impairments, learning difficulties, neurodevelopmental and neuropsychiatric disorders like Autism spectrum disorder (ASD), Schizophrenia (SCZ), Attention Deficit Hyperactivity Disorder (ADHD), or Obsessive–Compulsive Disorder (OCD) and could render children more prone to other health issues [25].

## 4. The Case of Autism

ASD is a developmental condition marked by ongoing challenges in social communication and interaction across different situations, together with restricted or repetitive behaviors, interests, or activities. These features usually emerge during early childhood, lead to notable difficulties in social, occupational, or other key areas of daily life, and cannot be solely attributed to intellectual disability or general developmental delay.

### 4.1. Definition, Epidemiology and Causes

Initially, the term “autism” was used to describe a behavioral symptom of SCZ. It was later redefined by Dr. Hans Asperger and Dr. Leo Kanner to describe a childhood syndrome characterized by social deficits and repetitive behaviors [26]. ASD now refers to a group of neurodevelopmental conditions, including Autism, Asperger’s syndrome, Pervasive Developmental Disorder–Not Otherwise Specified (PDD-NOS) and genetic syndromes with ASD traits such as Rett syndrome, Fragile X syndrome (FXS) and Tuberous Sclerosis Complex (TSC) [26,27,28,29]. ASD is among the leading causes of disability in children worldwide [25]. In 2019, there were an estimated 28.3 million prevalent cases globally, and 4.3 million DALYs (ASR: 56.3 per 100,000). From 1990 to 2019, prevalence and DALYs increased by nearly 40%, while incidence and ASRs remained relatively stable, partly due to changes in diagnostic criteria and tools [30].

As of 2022, about 1 in 31 children in the United States have been identified with ASD, though estimates vary across regions and study methods. Globally, Autism is thought to affect roughly 1 in 100 children, with considerable variation depending on diagnostic criteria and practices.

The clinical features that characterize ASD involve the triad of repetitive behaviors, restricted interests and differentiation in social interactions, and they are used for diagnosis since there are no diagnostic biomarkers [26]. ASD symptoms have been linked to various mental, physical, neurodevelopmental and neuropsychiatric disorders that can affect an individual’s and their family’s life quality during and after childhood [30]. ASD comorbidities include psychological, like ADHD, depression, OCD or physiological, like sleep disorders that exacerbate the severity of symptoms and epilepsy, which possibly appear because of similarities in brain pathologies like synaptic defects [26,31,32]. SCZ has also been associated with ASD since the two present similarities in risk factors, brain biochemistry and behavior [26]. Consequently, these divergent plasticity mechanisms manifest as early-onset social/cognitive differences in ASD versus adolescence-onset deficits in SCZ.

It is well known that a plethora of genetic and non-genetic factors contribute to the manifestation of ASD, although the cause is still unknown. Prenatal factors, developmental psychology, family history, environmental factors, immune system and genetics have been only some of the areas of interest of various studies [15,30]. ASD appears to be highly heritable with about 1000 associated genes presenting ASD-associated polymorphisms, genetic variations and de novo mutations in 20–25% of the cases [26]. The concordance rate for identical twins is 70–90% and fraternal twins is 0–10%. Non-genetic factors involve prenatal stress, parental age, maternal metabolic condition and mineral/ vitamin/nutrient intake, infections in pregnancy and contact with certain heavy metals, toxins or drugs like antiepileptics, antidepressants and valproate [26]. Moreover, maternal responses to infections by measles, rubella, cytomegalovirus and chicken pox [33] may lead to disproportionate inflammation and cytokine levels and increased cytokine levels in the fetus, affecting gene expression or even the immune system later in adulthood [26]. Additional activation of microglia and astroglia cells indicate a possible phenotype of neuroinflammation involved in ASD [26].

### 4.2. Neuropathology, Neurogenetics and Neurochemistry Protagonists

Regarding the structural characteristics of Autism, neuroanatomical studies have reported macrocephaly and abnormal neuronal connectivity, which seem to display localized overconnectivity and long-range or inter-regional underconnectivity, in ASD individuals [33]. However, early brain overgrowth is not universal, and considerable heterogeneity exists across individuals and subtypes, with some children showing normative or even reduced brain volumes [34]. Hyperplasia mainly affects the cerebral cortex, particularly the frontal and temporal lobes, which play key roles in higher brain functions such as social functioning and language development [33]. It has been suggested that ASD is associated with abrogation of the white matter tracts that are located in regions responsible for social cognition, like the anterior cingulate cortex, prefrontal cortex and superior temporal regions or in regions associated with language and working memory [33]. Alterations of gray or white matter have been detected in additional regions, like the frontoparietal regions, hippocampus, cingulate, amygdala, basal ganglia [29], cerebellum and subcortical limbic structures. Furthermore, there have been reports of hypoplasia of hemispheres and cerebellar vermis, as well as a decrease in Purkinje cells of the cerebellum and of cerebellar activation during selective attention tasks that is enhanced during simple motor tasks, a trait related to Autism [33]. These structural patterns may correspond to dysregulated excitatory/inhibitory balance, excessive spine density or impaired pruning in early-developing circuits, providing a mechanistic link between anatomical variability and functional outcomes in ASD. Thus, cellular changes reflect opposite plasticity patterns: increased dendritic spines and cortical overgrowth in ASD and reduced spines and pruning in SCZ.

Synaptic impairment, which is involved in various neurodevelopmental and neuropsychiatric disorders, appears to be the main neuropathology in ASD and is associated with cognitive and functional impairments. More specifically, FXS mutations in the FMR1 gene repress production of the FMR1 protein (FMRP) that is involved in mRNA translation and transport to synapses and dendrites, affecting the intellectual status and the presence of ASD features in individuals [29,33]. Notably, FMRP targets various ASD-associated molecules like mTOR, TSC2, NF1, Shank3, neurexin1 and neuroligin2, which are also associated with NMDAR and mGluR [33]. In Rett syndrome, mutations in the MECP2 gene on the X chromosome can lead to ASD, brain abnormalities, intellectual disability or death in males, while in TSC ASD, cognitive dysfunctions and brain tumors can be detected due to mutations in the TSC1 or TSC2 genes [29,33]. Additionally, neurotrophic factors, like BDNF, which was detected in high levels in ASD individuals, seem to also have a connection with ASD, as well as Reelin, which is associated with epilepsy and SCZ as well and plays a role in brain development and neuronal migration [33].

**Figure 1:**
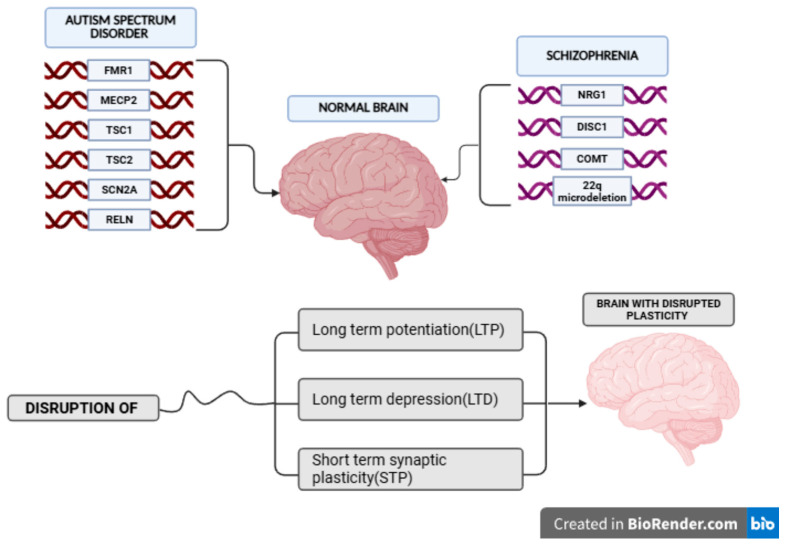
Conceptual diagram illustrates how distinct genetic risk factors for ASD and Schizophrenia converge mechanistically, resulting in a brain with disrupted plasticity. Genes associated with increased risk for ASD, including FMR1, MECP2, TSC1, TSC2, SCN2A and RELN, are shown on the left. Key genetic risk factors for Schizophrenia, including NRG1, DISC1, COMT and the 22q microdeletion, are shown on the right. Both groups of genetic factors are hypothesized to influence the developing of normal brain state. The model proposes that the primary pathological outcome is the disruption of fundamental mechanisms of synaptic plasticity: Long Term Potentiation (LTP), Long Term Depression (LTD) and Short-Term Synaptic Plasticity (STP). This disruption of synaptic function ultimately leads to the final, pathological state, characterized as a brain with disrupted plasticity, suggesting a shared final common pathway in the pathophysiology of these two major neurodevelopmental disorders. Created in BioRender. Kesidou, E. (2026) https://BioRender.com/8jga3o6 (Accessed on 3 September 2025).

ASD has been linked to an abnormal GABAergic system and diverse glutamate receptors. Abnormal levels of glutamate and glutamine have been detected in the plasma of children with Autism [33]. Furthermore, studies on synaptogenesis abnormalities have shown that 17 out of 107 genes involved in synaptic formation, plasticity and homeostasis are also common in studies on patients with SCZ. Such genes are SHANK3 of the SHANK family with postsynaptic scaffolding proteins, NRXN1 a neurexin of the cell adhesion family, neuroligins [26], SCN2A and RELN, which are involved in glutamatergic synaptic transmission, with the first two being also involved in ASD and synapse formation and maturation [29,33] (Figure 1). The two important signaling pathways in ASD pathology are the NRXN-NLGN-SHANK and mTOR/PI3K pathway [26], which take part in the regulation of synaptogenesis. Disruptions in these pathways alter AMPAR and NMDAR trafficking, leading to abnormal dendritic spine density and impaired synaptic scaling, and may perturb the timing of critical periods for circuit refinement. Evidence from animal models and iPSC-derived neurons indicates that NRXN/NLGN/SHANK mutations reduce synaptic maturation and plasticity, whereas mTOR/PI3K dysregulation enhances excitatory synapse formation, contributing to hyperconnectivity and the excitatory/inhibitory imbalance characteristic of ASD [35]. Together, these molecular alterations produce early hyperplasticity in ASD versus adolescent hypo/dysplasticity in SCZ.

### 4.3. Synaptic and Non-Synaptic Plasticity in Autism

Core behaviors in ASD are linked to atypical connectivity between higher-order association areas, including the dorsolateral prefrontal cortex and anterior cingulate cortex [36]. While the underconnectivity hypothesis emphasizes reduced long-range connectivity, local hyperconnectivity often coexists, especially in sensory and frontal circuits [37]. ASD emerges in early childhood, during rapid synaptogenesis and network maturation, before substantial pruning. Some frontal and temporal networks may experience early closure of heightened plasticity windows, though this “premature closure” remains a partially supported hypothesis. Synapse formation and dendritic spine expansion underlie experience-dependent circuit refinement, highlighting both hyper- and hypo-connectivity in ASD. Circuit-level disruptions mirror these trends: ASD shows local hyper- and long-range underconnectivity early, while SCZ exhibits adolescent long-range disconnection.

During the postnatal period, there is a notable expansion of short-range cortico-cortical pathways, which likely plays a role in the extensive reorganization of cortical circuitry and synaptic functions [38]. The early rise in primary network connectivity also corresponds with a partial reduction in certain connections [39]. Evidence suggests that the posterior cingulate/retrosplenial cortex serves as a primary neural hub, while the medial prefrontal cortex may emerge as a secondary hub starting around one year of age. In this early developmental window, cortical activity is predominantly shaped by environmental input and gradually shifts toward experience- and sensory-dependent processes. This transition is underpinned by increasing myelination [40]. As infants approach the latter half of their first year, social stimuli gain significance and begin to influence the organization of socially relevant cortico-cortical networks [41].

Taken together, this evidence indicates that key developmental processes may be altered within the first two years of life, potentially impacting synaptic activity in specific cortical networks. Such alterations may constitute a “second hit” in the developmental course of ASD. Early abnormalities in synaptogenesis, combined with the heightened susceptibility of limbic and lateral neocortical regions during prenatal development and subsequent reorganization in the second postnatal stage, are thought to contribute to atypical connectivity patterns, including overconnectivity, hypoconnectivity and dysconnectivity [41]. Disruptions in connectivity have been reported both within the frontal lobe and between the frontal and temporal regions, observed at rest as well as during tasks involving face recognition, sentence comprehension and emotional expression. These findings are consistent with the hypothesis of frontal–posterior underconnectivity in ASD [42,43].

Rubenstein proposed in 2003 that an elevated ratio of cortical excitation to inhibition (E/I) may underline certain forms of ASD, leading to cortical hyperexcitability [44]. This E/I imbalance (favoring excitation), driven by synaptic adhesion anomalies, mTOR/SHANK pathway alterations and impaired inhibitory regulation, disrupts plasticity homeostasis and drives sensory hyper-responsivity [45]. More recent reviews have identified disruptions in GABAergic and glutamatergic neurotransmission within key brain regions as contributors to this E/I imbalance in ASD [46], which may both influence and result from network overconnectivity. The concept that a finely tuned E/I balance is essential for critical period plasticity suggests that disturbances in this balance could underline a range of ASD-related neural alterations. Since the maturation of frontal networks appears to be highly dependent on experience-driven input during a critical plasticity period, typically around ages 2 to 3, premature closure of this window may heighten the vulnerability of these networks in ASD. Notably, frontal brain networks exhibit the most prolonged developmental trajectory among all brain regions [37,47]. This stands in contrast to other neural circuits that remain relatively unaffected in ASD [48], suggesting that some functions may be less reliant on extrinsic, experience-dependent plasticity mechanisms [36]. In general, in ASD there may be premature closure of plasticity windows affecting critical periods and developmental windows marked by heightened plasticity, which are essential for the wiring of sensory, language and social circuits. Evidence supports early closure of these periods, truncating experience-dependent plasticity and constraining circuit refinement [49].

## 5. The Case of Schizophrenia

### 5.1. Epidemiology and Stimuli

SCZ is a severe psychiatric disorder marked by impairments in basic cognitive functions such as learning, emotional regulation and perception [50]. Its clinical features include: (i) negative symptoms like anhedonia, apathy, social withdrawal, alogia and blunted affect; (ii) positive symptoms such as hallucinations, delusions and disorganized behavior [51]; and (iii) cognitive deficits impacting attention, executive function and memory [52].

SCZ affects about 24 million people worldwide, or 0.32% of the global population (around 1 in 300). Among adults, the rate is higher (approximately 0.45%, or 1 in 222). SCZ is a multifactorial mental health disorder shaped by genetic, neurodevelopmental, environmental and psychological influences. A family history, particularly a first-degree relative with the condition, is one of the strongest risk factors. Certain gene variants, such as COMT, DISC1 and NRG1, are also linked to increased susceptibility [53]. Neurodevelopmental risks include prenatal exposure to infections (e.g., influenza, toxoplasmosis) and malnutrition during early gestation.

Environmental factors significantly influence SCZ risk. Urban upbringing is linked to higher incidence compared to rural environments. Cannabis use, especially in adolescence and in genetically susceptible individuals, also increases risk. Prenatal infection or malnutrition may prime microglia and complement pathways, predisposing circuits to excessive adolescent pruning. Structural and neurochemical brain abnormalities, such as enlarged ventricles, reduced gray matter and dopamine and glutamate imbalances, are commonly observed. Abnormal synaptic pruning during adolescence, which may disrupt plasticity [54] and impair neuroplasticity, both structural and synaptic, is a major contributor to the disorder’s development [53].

### 5.2. Synaptic and Non-Synaptic Plasticity in Schizophrenia

Evidence suggests that disruptions in early brain development, affecting neurogenesis, neural migration and synaptogenesis [55], as well as in later processes like synaptic pruning [56], contribute to SCZ. Both reduced and abnormally excessive plasticity may underline its negative and positive symptoms. Additionally, dysfunctions in NMDARs and glutamatergic circuits are implicated in psychotic and cognitive symptoms [57]. Abnormalities in GABAergic pathways also impair plasticity, with reduced GABAergic activity potentially decreasing cortical plasticity [58]

**Figure 2:**
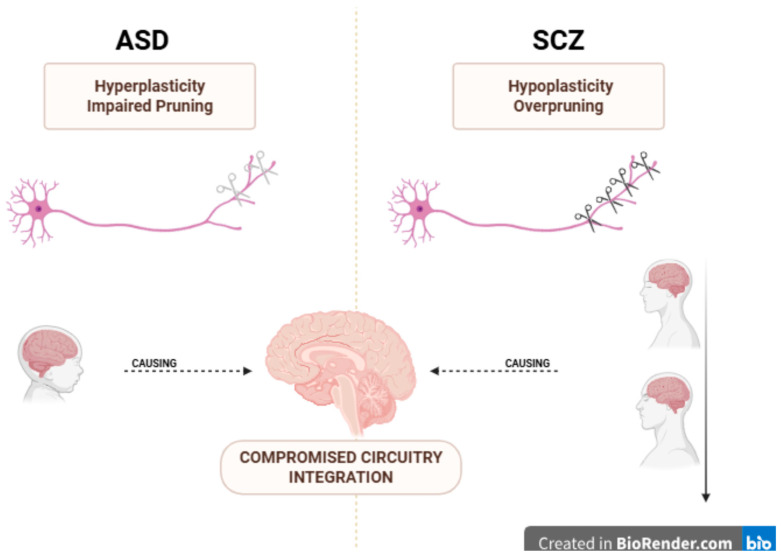
This diagram illustrates the divergent hypotheses regarding synaptic pruning abnormalities in two major neurodevelopmental disorders. On the left, ASD is associated with hyperplasticity and impaired pruning, visually represented by a neuron with excessive, uncleared synaptic connections and a scissor icon indicating inhibited pruning. This suggests an overabundance of synapses, potentially leading to neural “noise” or inefficiency. On the right, SCZ is linked to hypoplasticity and overpruning, depicted by a neuron with an exaggerated loss of synaptic connections, where a scissor icon actively removes dendritic branches. This implies an excessive elimination of synapses, potentially leading to sparse or dysfunctional neural networks. Both mechanisms, whether under-pruning or overpruning, are proposed to result in a central pathological outcome: compromised circuitry integration within the brain. Created in BioRender. Kesidou, E. (2026) https://BioRender.com/4n4uc0c (Accessed on 3 September 2025).

Another aspect of dysplasticity in SCZ is the abnormal myelination mediated by oligodendrocytes [59] and the augmented activity of microglia in the frontal cortex and the hippocampus, as imaging and post mortem studies have indicated [60]. Specifically, microglial activation shows polarization in SCZ. A study conducted in Japan [61] indicated that M1 (pro-inflammatory type) polarization of microglia has been associated with relapses as a result of the disruption in neural networks. On the other hand, M2 (anti-inflammatory) polarization has been associated with remission because of the inhibitory effects on M1 microglia. However, TSPO-PET findings are inconsistent, and the M1/M2 framework oversimplifies microglial states; therefore, cell-type resolved human studies are needed. SCZ has been conceptualized as a disorder of overpruning: elevated microglial activity, complement-mediated synapse elimination (notably involving C4 and C3) and reduced dendritic spine density are implicated [62] (Figure 2). In SCZ, critical period machinery appears to remain open or improperly regulated into adolescence. Altered maturation of parvalbumin interneurons, delayed perineuronal net formation and extended circuit susceptibility contribute to circuit instability and psychosis risk [49]. Recent electrophysiological and postmortem studies indicate that impaired activity-dependent plasticity of parvalbumin-positive interneurons in the dorsolateral prefrontal cortex disrupts gamma oscillatory synchrony, compromising local circuit integration and working-memory-related network stability in SCZ [63]. SCZ pathophysiology has also been linked to NMDA receptor hypofunction, leading to plasticity deficits (impaired LTP/LTD) and GABAergic impairments that hinder cortical inhibition. These neurochemical disruptions are detectable via impaired NIBS-induced cortical plasticity and are central to cognitive and negative symptoms [64]. Consistent with these findings, TMS and EEG studies have shown reduced LTP-like and LTD-like plasticity in the dorsolateral prefrontal cortex of SCZ patients, correlating with deficits in executive function and cognitive flexibility [65].

Genetic abnormalities affecting neurotrophin expression, BDNF, have emerged as highly significant. BDNF plays a critical role in modulating dopaminergic neurotransmission by influencing the expression of D1 and D5 dopamine receptors. It is also thought to contribute to the potentiation of synaptic responses to tetanic stimulation [66]. Research has demonstrated that individuals with SCZ exhibit lower BDNF levels compared to healthy controls [67].

The comparison of motor evoked potentials (MEP) and motor threshold (MT) prior to and after repetitive transcranial magnetic stimulation (rTMS) can be used to examine the changes that LTPs and LTDs demonstrate in SCZ patients. Both LTP and LTD are involved in memory formation and learning, and several studies have shown that conventional MEP changes are not exhibited in LTD and LTP plasticity in patients with SCZ [68,69]. However, these findings are derived from small samples with heterogeneous protocols and effects on clinical outcomes that remain modest and variable; standardized stimulation protocols and coupling of physiological measures to functional endpoints are needed.

### 5.3. Genetic Predisposition

Several genetic studies over the last decades have shown that SCZ has high heritability and that numerous gene alterations can lead to the effects of the disorder. These genes encode factors that are responsible for the regulation of synaptic transmission (glutamergic, GABAergic, dopaminergic), brain maturation and cell proliferation and have significant roles in brain development and plasticity [70]. The protein neuregulin 1, which is encoded by the gene NRG1, is a neurotrophic protein that is essential for the neurogenesis (multiplication) of the hippocampus and the cortex–amygdala circuits (synaptic plasticity) [71]. Studies have shown that heterozygous deletion of NRG1 leads to disruption of theta burst LTP in the formation of the hippocampus [72]. The gene which was first associated not only with SCZ, but several other mental conditions too, is DISC1 [73]. It is proved that DISC1 conjugates with Pbe4b and Gsk3β, proteins that are known to take part in the pathology of SCZ. In mice, mutations on the DISC1, such as the mutation L100P, can cause the gene to undergo an abnormal interaction with these factors and consequently, lead to disrupted working and object recognition memory [74]. Another crucial fact about DISC1 is that postnatally, it regulates the maturation of dopaminergic and GABAergic synaptic transmission, especially in the prefrontal cortex and that the modification of the gene at specific time points has shown to alter this maturation [75].

The 22q11 microdeletion syndrome is recognized as a significant genetic risk factor for various cognitive disorders, including SCZ [76]. Animal model studies investigating the relationship between genetic alterations and neuroplasticity have demonstrated that 22q11 deletion is associated with impaired or dysregulated synaptic plasticity, aberrant synaptic transmission and disrupted neuronal maturation and differentiation from prenatal through to postnatal development. Notably, alterations in the ZDHHC8 gene, identified as a SCZ susceptibility gene, have been shown to reduce synaptic strength in both the hippocampus and cerebral cortex, as well as to induce abnormal axonal branching and growth.

A few genetic risk factors for SCZ, including NRG1, DISC1 and 22q11 microdeletions, converge on synaptic development, plasticity and the refinement of circuitry [77]. Interestingly, several of these genes (including, among others, SHANK3, NRXN1 and the neuroligins) are also implicated in ASD, indicating common molecular substrates. However, there are divergences in terms of the developmental time course; in ASD they affect early postnatal synaptogenesis and local circuitry formation, resulting in hyperplasticity and excitation–inhibition imbalance, whereas in SCZ the same or overlapping genes influence adolescent pruning and refinement of synapses, leading to hypo- or dys-plasticity. This temporal distinction may underlie the contrasting phenotypes and critical-period vulnerabilities observed in the two disorders.

## 6. Rehabilitation

Given the fact that neuroplasticity plays a major role in the evolution of a plethora of neurological, neurodegenerative and neurodevelopmental disorders, such as brain injury and stroke, the use of strategies in order to stimulate neuroplasticity pathways could possibly restore cognitive and behavioral deficiencies caused by inefficient plasticity or dysplasticity.

There is plenty of evidence that antipsychotic medication contributes to many beneficial molecular and structural changes. Medication and especially antipsychotics have been the first line of treatment of SCZ. For instance, neuroimaging studies have proven that antipsychotic medication is linked to augmentation of the striatum and other structures located in the basal ganglia, [72,73], thalamus and in the gray matter of the cortex [74].

However, in the cases of SCZ and ASD, a line of studies indicates that rehabilitation, by using the neuroplastic properties of the brain, is possible and the results of these studies are promising. Especially in children, neuroplasticity is much more modifiable. Rehabilitation consists of several methods, such as cognitive training, physical exercise, non-invasive brain stimulation and, more recently, virtual reality, which has also shown promising evidence that brain plasticity can be harnessed for therapeutic purposes. For SCZ, cognitive rehabilitation in small trials (n < 50) has been associated with modest increases in gray matter thickness in the hippocampus and amygdala [78], normalization of interhemispheric connectivity via the corpus callosum [79] and elevated serum BDNF [80], though replication and long-term durability are limited. For ASD, pilot studies indicate that targeted cognitive or VR-based interventions may improve executive functions and social skills, but trials are small, heterogeneous and outcomes vary by intervention type and age.

Another strategy that uses brain plasticity to treat symptoms of SCZ is non-invasive brain stimulation. It is conducted by targeting regions of the brain that are proved to have deficiencies and hence cause negative, positive or cognitive symptoms. The protocol until now consists of targeting specific areas that are biologically proven to have deficiencies with high- or low-frequency TMS pulses. A meta-analysis showed that low-frequency rTMS applied to regions such as the left temporoparietal cortex produces modest, domain-specific improvements in auditory hallucinations [81]. Although noninvasive brain stimulation may modestly affect symptom trajectories, results vary with target, frequency and sham condition and its practice remains experimental.

A further non-invasive approach that concerns the improvement of SCZ symptoms is physical exercise that causes the blood flow to and oxygenation levels of the hippocampus and the BDNF to increase [82], and this environmental enrichment can boost neural circuits.

We have discussed several factors that play a significant role in the symptomatology of SCZ and some of the strategies that can be conducted in order to harness the aberrant hyperplasticity and evoke the plasticity of brain regions and neuronal circuits that are incompletely developed. Overall, the understanding of the molecular mechanisms that are crucial to the manifestation of SCZ would lead to developing new effective therapeutic strategies.

Cognitive rehabilitation strategies have recently been implemented to target Executive Functions (EF) and related cognitive processes such as working memory, motor and social skills in children with Specific Learning Difficulties (SLD), including ASD and ADHD. Pilot studies and small trials suggest that, in addition to pharmaceutical treatment, non-invasive methods, such as Virtual Reality (VR), Forehead Lobe Exercise Program (FEP, a locally developed EF training paradigm) and non-invasive brain stimulation, may modestly influence cognitive outcomes.

Specifically, a pilot VR study targeting EFs in children with SLD reported improvements in visual attention, inhibition, flexibility and planning skills, sustained for six months [83]. A protocol for a clinical tDCS trial in children with ASD was designed to apply stimulation to motor-related regions (primary motor cortex and cerebellum) during motor training; efficacy remains to be tested [84]. Lastly, a small FEP pilot study indicated potential gains in working memory, flexibility and planning in children with High-Functioning ASD [85], though replication and standardization are needed [86].

## 7. Limitations

This review is limited by the predominance of preclinical and small clinical studies, heterogeneous interventions and variable outcome measures. Direct links between molecular plasticity changes and clinical effects remain largely inferred. Larger, longitudinal trials are needed to confirm findings and clarify causal relationships. Individual variability and overlap with other neurodevelopmental conditions may also influence observed patterns.

## 8. Future Directions

Future research should develop both developmentally and circuit-specific treatments that can take advantage of neuroplasticity in ASD and SCZ. Important questions in this area are to understand how to connect synaptic/molecular changes to improved behavioral outcomes, what the relevant biomarkers are to develop personalized therapy and how genetic or excitation/inhibition (E/I) profiles may reflect treatment response. Large multi-site studies providing cognitive, neuroimaging and neurophysiological measurements are needed to validate and optimize treatments.

## 9. Conclusions

Neuroplasticity provides the foundation for the brain’s ability to adapt and reorganize based on internal and external influences and is necessary for neurodevelopment, learning and recovery. Dysregulation of synaptic and non-synaptic plasticity has been strongly implicated in the pathophysiology of neurodevelopmental disorders such as ASD and SCZ, both of which have characteristic alterations of synaptic connections and neurotransmission. ASD and SCZ could be conceptualized as poles of a neuroplasticity dysregulation continuum (precocious hyperplasticity versus delayed/dysregulated hypo/dysplasticity) while acknowledging considerable heterogeneity within each diagnosis and partial overlap of phenotypes; testable predictions could be pursued using TMS-LTP/LTD measures and MRS assessment of Glu/GABA balance. Notably, despite conceptualizing these disorders at opposite ends of the continuum, co-occurrence can still occur, underscoring the need to consider shared vulnerabilities and overlapping phenotypes. This may be explained because of common genetic and epigenetic mechanisms, such as risk loci overlap in genes related to synaptic and neurotransmission molecules and histone acetylation and methylation that are linked to maternal infections and perinatal and postnatal hypoxia. Exposure of the molecular and structural processes behind plasticity provides important insights into these disorders and identifies potential therapeutic avenues. Emerging rehabilitation strategies—cognitive training, non-invasive brain stimulation, drug treatment and virtual reality-based therapy—hold the ability to engage and enhance neuroplasticity, with promising avenues for boosting cognitive and behavioral gains. Further study of developmentally timed, individually tailored and precision-guided treatments holds promise to optimize effects. Future research should focus on developmentally targeted trials (ASD: early childhood; SCZ: prodrome/adolescence), circuit-specific interventions and biomarker-guided approaches (TMS plasticity metrics, MRS Glu/GABA, EEG indices). Stratification by genetic profile or E/I phenotype and multi-site replication will be essential to validate and generalize findings across neurodevelopmental disorders.

## Figures and Tables

**Table 1:** Comparative overview of neuroplasticity dimensions in ASD and SCZ. The table summarizes key differences in the direction and developmental timing of plasticity alterations across core neurobiological dimensions, including synaptic plasticity, connectivity, critical periods, excitation–inhibition balance and microglial-mediated pruning. Representative molecular pathways, physiological and imaging markers and therapeutic implications are highlighted for each domain.

Dimension	ASD (Typical Direction/Timing)	SCZ (Typical Direction/Timing)	Key Molecular Nodes	Phys/Imaging Markers	Therapeutic Implications
Synaptic plasticity	Early hyperplasticity; excess spine formation in early childhood	Hypoplasticity/overpruning in adolescence	FMRP, mTOR/PI3K, SHANK3 (ASD); NRG1, DISC1, C4/C3 (SCZ)	Spine density, dendritic morphology (postmortem/iPSC), MRI cortical thickness	Early cognitive training, EF interventions (ASD); rTMS, cognitive remediation (SCZ)
Connectivity	Local hyperconnectivity, long-range underconnectivity; early critical period	Reduced long-range connectivity; delayed circuit refinement	NRXN/NLGN/SHANK, GABA/glutamate receptors	DTI, fMRI resting-state networks	Circuit-targeted VR, behavioral therapies (ASD); network-guided rTMS (SCZ)
Critical periods	Early sensory/social circuits (0–3 years)	Adolescent refinement (12–25 years)	GABAergic maturation, parvalbumin interneurons	MRS GABA/Glu, EEG/ERP	Developmentally timed interventions; experience-dependent plasticity harnessing
Excitation/inhibition balance	E/I shift favoring excitation → hyper-responsivity	E/I shift favoring reduced inhibition → network instability	GABA, Glutamate, mTOR, SHANK, NMDAR	MRS Glu/GABA, TMS plasticity metrics	Pharmacological modulation, activity-dependent interventions
Microglia/pruning	Impaired pruning → excess early connections	Excess pruning → synapse loss, network dysregulation	Complement (C3/C4), microglia activation	TSPO-PET, postmortem histology	Anti-inflammatory approaches; precision-timed interventions

## Data Availability

No new data were created or analyzed in this study. Data sharing is not applicable to this article.

## Abbreviations

AMPAR	α-Amino-3-hydroxy-5-methyl-4-isoxazolepropionic Acid Receptor
ASD	Autism Spectrum Disorder
ASR	Age-Standardized Rate
DALYs	Disability-Adjusted Life Years
EEG	Electroencephalography
E/I	Excitation/Inhibition
EF	Executive Functions
FEP	Forehead Lobe Exercise Program
LTD	Long-Term Depression
LTP	Long-Term Potentiation
MRS	Magnetic Resonance Spectroscopy
NMDAR	*N*-Methyl-d-aspartate Receptor
rTMS	Repetitive Transcranial Magnetic Stimulation
SCZ	Schizophrenia
SLD	Specific Learning Difficulties
STP	Short-Term Plasticity
tDCS	Transcranial Direct Current Stimulation
VR	Virtual Reality
